# Single-blind randomized clinical trial to evaluate clinical and radiological outcomes after one year of immediate versus delayed implant placement supporting full-arch prostheses

**DOI:** 10.4317/medoral.19536

**Published:** 2013-12-07

**Authors:** Hilario Pellicer-Chover, David Peñarrocha-Oltra, Leticia Bagán, Antonio J. Fichy-Fernandez, Luigi Canullo, Miguel Peñarrocha-Diago

**Affiliations:** 1 Master in Oral Surgery and Implant Dentistry, Faculty of Medicine and Dentistry, University of Valencia, Spain; 2 Collaborator of Oral Medicine, Stomatology Department, Faculty of Medicine and Dentistry, University of Valencia, Spain; 3 Master in Oral Surgery and Implant Dentistry. Collaborating Lecturer on the Master in Oral Surgery and Implant Dentistry. Faculty of Medicine and Dentistry, University of Valencia, Spain; 4 In Private Practice in Rome. Collaborating Lecturer on the Master in Oral Surgery and Implant Dentistry, Valencia University Medical and Dental School, Spain; 5 Chairman of Oral Surgery and Director of the Master in Oral Surgery and Implant Dentistry, Faculty of Medicine and Dentistry, University of Valencia, Spain

## Abstract

Purpose: To evaluate and compare peri-implant health, marginal bone loss and success of immediate and delayed implant placement for rehabilitation with full-arch fixed prostheses. Material and Methods: The present study was a prospective, randomized, single-blind, clinical preliminary trial. Patients were randomized into two treatment groups. In Group A implants were placed immediately post-extraction and in Group B six months after extraction. The following control time-points were established: one week, six months and twelve months after loading. Measurements were taken of peri-implant crevicular fluid volume, plaque index, gingival retraction, keratinized mucosa, probing depth, modified gingival index and presence of mucositis. Implant success rates were evaluated for the two groups. The study sample included fifteen patients (nine women and six men) with a mean average age of 63.7 years. One hundred and forty-four implants were placed: 76 placed in healed sites and 68 placed immediately. Results: At the moment of prosthetic loading, keratinized mucosa width and probing depth were higher in immediate implants than delayed implants, with statistically significant differences. However, after six and twelve months, differences between groups had disappeared. Bone loss was 0.54 ± 0.39 mm for immediate implants and 0.66 ± 0.25 mm for delayed implants (p=0.201). No implants failed in either group. Conclusions: The present study with a short follow-up and a small sample yielded no statistically significant differences in implant success and peri-implant marginal bone loss between immediate and delayed implants with fixed full-arch prostheses. Peri-implant health showed no statistically significant differences for any of the studied parameters (crevicular fluid volume, plaque index, gingival retraction, keratinized mucosa, probing depth, modified gingival index and presence of mucositis) at the twelve-month follow-up.

** Key words:**Immediate implants, delayed implants, peri-implant health, success rate.

## Introduction

The immediate insertion of implants in post-extraction sockets is a treatment modality with high success rates (1). Chen *et al.* (2) conducted a literature review of success rates and clinical outcomes associated with immediate, early and delayed implant placement, finding similar success rates among the different procedures. Nevertheless, immediate placement has certain advantages over delayed implant insertion: the reduction in treatment time and the avoidance of second surgery (3).

It is thought that the long-term success of dental implant treatment depends on many factors, including periodical maintenance and follow-up examinations (4). However, few long-term controlled follow-up studies of implants that include periodontal pa-rameters have been reported. Salvi *et al.* (5) conducted a literature review and concluded that the parameters that may be applied for assessing the state of peri-implant health and the severity of peri-implant disease include: plaque accumulation, probing depth, bleeding on probing, keratinized mucosa width and crevicular fluid volume.

According to a recent literature review (6), only two randomized studies (7,8) have compared one-piece implants placed immediately following extractions with the same placed in healed sites. No controlled studies have been found that evaluate the influence on peri-implant health of placing implants immediately or after allowing socket healing in patients rehabilitated with fixed full-arch prostheses. In this way, the aim of this prospective controlled clinical trial was to evaluate and compare peri-implant health, marginal bone loss and success of immediate and delayed implants rehabilitated with full-arch fixed prostheses.

## Material and Methods

* Study Population

The present study was a prospective, randomized, single-blind, clinical preliminary trial carried out at the Oral Surgery Unit of the University of Valencia between December 2009 and February 2011 on patients requiring implant-supported fixed full-arch prosthetic rehabilitations. Stratification was performed considering the arch to be treated (maxilla/mandible). Random group assignment was performed by a professional statistician using pre-defined randomization tables. A balanced random permuted-block approach was used to prepare the randomization tables in order to avoid unequal balance between the two treatment groups.

Patients received eight implants in the maxilla and/or six implants in the mandible to support fixed full-arch prosthetic rehabilitations. Group A patients received implants immediately after extraction; any non-immediate implants in Group A were excluded from analysis. In Group B, all implants were placed in healed sites; the necessary dental extractions were performed during the six months preceding implant placement.

This research was performed following recommendations made in the Consort Statement for reporting randomized clinical trials and the principles of the Declaration of Helsinki regarding research on humans; accordingly, all patients provided written informed consent to take part in the trial prior to surgery. The study design was approved by the ethical board of the University of Valencia (Ref. H1335344377076).

* Selection Criteria

Inclusion criteria for this study were:

- Age > 18 years

- Full mouth plaque score and full mouth bleeding score < 25 %.

- Partially edentulous maxilla with indications for the extraction of all remaining teeth.

- Final rehabilitation with fixed full-arch implant-supported prosthesis.

- Sufficient bone height and width to place six to eight implants with a minimum length of 10 mm and minimum diameter of 3.8 mm without performing bone grafting procedures (sinus lifting, block bone grafts or GBR); coverage of peri-implant defects and/or gap filling with autologous bone or tricalcium ß-phosphate did not prevent inclusion in the study.

- Patients receiving six or more implants with insertion torque > 35 Ncm.

- For immediate post-extraction implants, > 4 mm of apical bone were required to ensure the necessary primary stability.

- Signature of informed consent form.

- Minimum follow-up of 12 months after implant loading.

* Exclusion criteria were:

- Sites with acute infection

- Implants placed with insertion torque < 35 Ncm were excluded from both groups.

- Medical conditions contraindicating implant surgery.

- Pregnant and lactating patients.

- Smokers.

- Patients with a history of bisphosphonate therapy.

- Patients receiving chemo or radiotherapy of head and neck.

- Severe bruxism.

- Poor oral hygiene or non-collaborative patients.

- Incomplete data gathering or failure to attend scheduled control appointments.

* Surgical Procedure

All surgeries were performed under local anesthesia (4% articaine with 1:100.000 adrenalin [Inibsa®, Lliça de Vall, Barcelona, Spain]). In Group B, non-immediate implants were placed following the standard surgical procedure. Periodontal treatment was delivered to Group A patients in order to control inflammation before performing the extractions. In Group A, anterior maxillary dental immediate implants were placed palatally, while for upper molars and premolars with two roots, implants were placed in the palatine root. In all maxillary cases, drills and osteotomes were used in combination to prepare implant beds. In the mandibular posterior area, implants were placed in the interradicular septum, whenever possible.

Implants used in the present study were Kohno SP® (Sweden&Martina, Due Carrare, Italy) with Dual Engineered Surface (DES®). All patients were treated following a one-step procedure. After implant placement and suturing, each patient received 500mg of amoxicillin (Clamoxyl, GlaxoSmithKline, Madrid, Spain) to be taken three times daily for seven days, 600mg ibuprofen (Bexistar, Laboratorio Bacino, Barcelona, Spain) to be taken as needed and a 0.12% chlorhexidine mouthwash (GUM, John O Butler/Sunstar, Chicago, IL, U.S.A.) for use twice daily for two weeks. Gentle brushing with a chlorhexidine tooth-paste was also recommended. Sutures were removed eight to ten days after surgery. Prosthetic loading was carried out after twelve weeks fol-lowing implant placement in the maxilla and after ten weeks in the mandible.

* Data Compilation and Follow-up

A previously established, standard protocol was used to compile the following data for all patients: patient age (at implant place-ment), sex, implant length and diameter.

Control visits were conducted by a trained clinician, blinded to group assignation, at the following time points: one week after prosthetic placement (time point 1); six months after loading (time point 2); and twelve months after loading (time point 3). At each time point the following data and clinical parameters were registered:

• Crevicular Fluid Volume (CFV): each sample was taken using the technique described by Offenbacher *et al.* (9).

• Gingival Retraction: was determined as being either present or absent and where present was measured in millimeters from the midfacial mucosa level to the restorative crown margin (10).

• Plaque Index (PI): the modified PI according to Mombelli *et al.* (11) was used: score 0 = no plaque; score 1 = plaque only detected with probe; score 2 = plaque visible to the naked eye; score 3 = abundant plaque.

• Probing Depth (PD): a periodontal probe (Click-Probe®, Kerr, Bioggio, Switzerland) was used to measure PD.

• Keratinized Mucosa (KM): utilizing the same probe, this was measured in millimeters from the mucogingival junction to the most coronal point of the keratinized mucosa at the center of the prosthetic restoration.

• Modified Gingival Index (mGI): the modified gingival index according to Mombelli *et al.* (11) was used: score 0 = no bleeding; score 1 = isolated bleeding spots; score 2 = confluent blood; score 3 = profuse bleeding.

• Mucositis: an implant was considered to have mucositis when reversible inflammation of the peri-implant mucosa without bone loss existed. The most important diagnostic factor for this pathology was bleeding on probing with a maximum force of 0.25N (12).

The definition of implant success was based on the clinical and radiographic criteria defined by Buser *et al.* (13): 1) absence of clinically detectable implant mobility; 2) absence of pain or any subjective sensation; 3) absence of recurrent peri-implant infec-tion; 4) absence of ongoing radiolucency around the implant after six and twelve months of loading.

**Figure 1:**
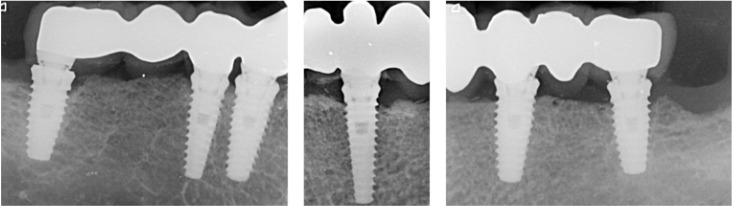
Radiographic assessment at the 3-month follow-up.

**Figure 2:**
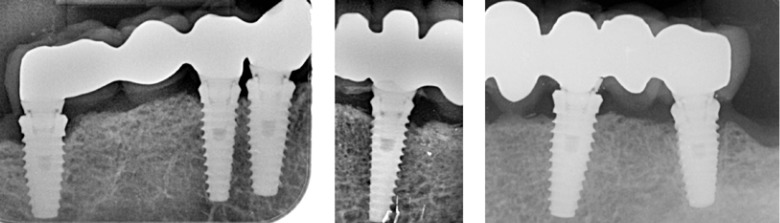
Radiographic assessment at the 12-month follow-up.

Intraoral radiographs were made at prosthetic loading (baseline) (Fig. 1) and at the one-year control (Fig. 2) visit using the XMIND intraoral system (Groupe Satelec-Pierre Rolland, Merignac, France) and an RVG intraoral digital receptor (Dürr Dental, Bietigheim-Bissingen, Germany) with the aid of Rinn XCP (Dentsply Rinn, Elgin, IL, U.S.A.) to achieve parallelism. If the bone level around the study implants was not clearly visible a new radiograph was made. The distance from the implant abutment connection to the peri-implant marginal bone level was measured to the nearest 0.1 mm both mesially and distally. Bone loss was calculated from the change in bone level between the baseline and the one-year control radiograph.

* Statistical Analysis

Clinical Parameter descriptive data were analyzed. Parametric test assumptions were checked and whenever there was doubt that these had been fulfilled, the corresponding non-parametric test was applied. All statistical analysis was performed using SPSS 15.0 for Windows (SPSS Inc, Chicago, IL, U.S.A.). Statistical significance was taken as 5% (*p*<0.05).

## Results

Patient Population

Of the 16 patients enrolled in this study, eight belonged to Group B (five maxillary and six mandibular) and eight to Group A (six maxillary and five mandibular). One patient in Group B, who had received six implants in the mandible, was excluded due to failure to attend control visits. The final study sample comprised 15 patients (six women and nine men) with a mean age of 63.7 years ( Table 1).

**Table 1:**
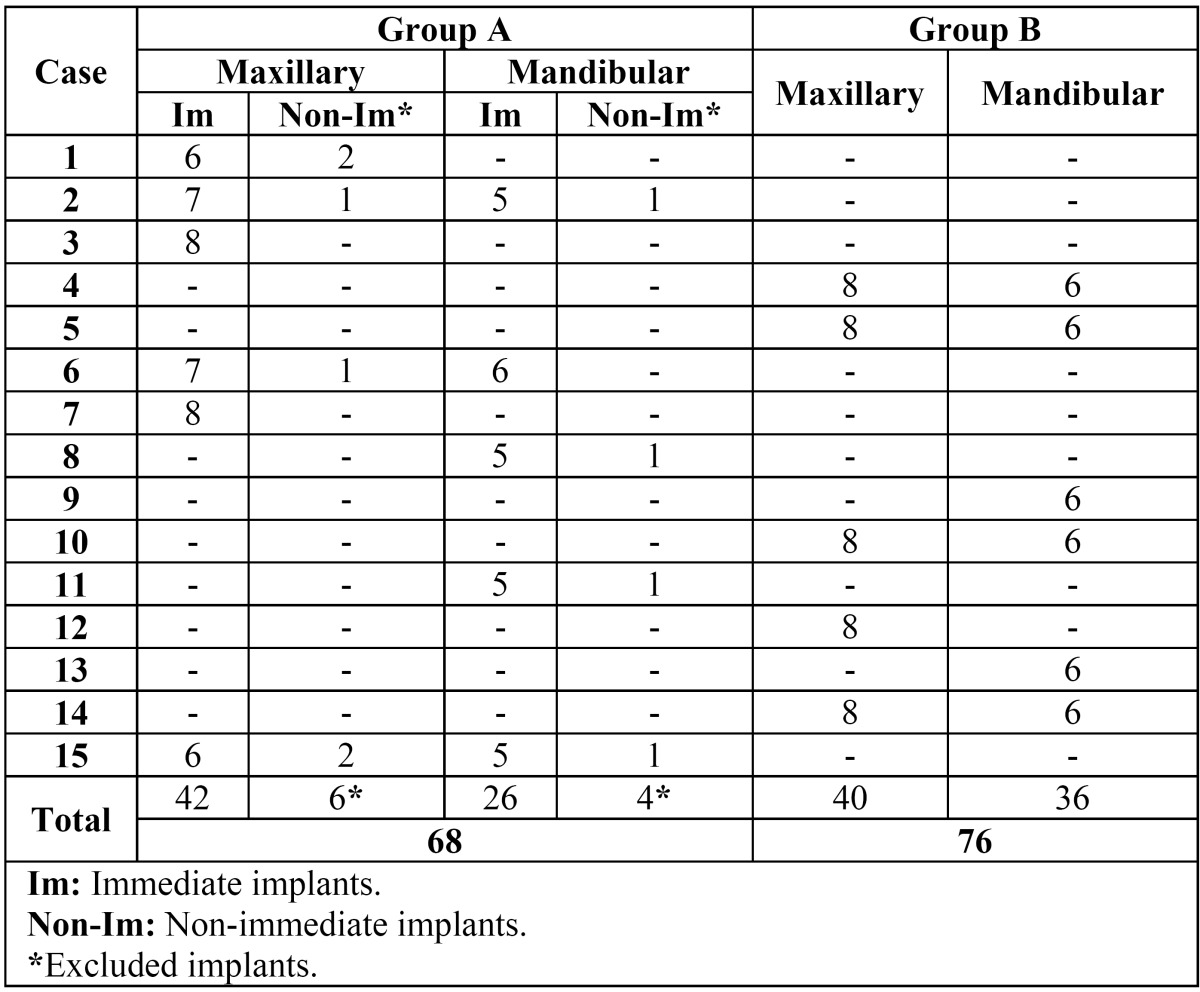
Patient and implant distribution per group.

Group B patients received 76 implants and Group A patients 68; ten non-immediate implants from Group A were excluded. In this way, a total of 144 implants were included in the study: 76 placed in healed sites and 68 placed immediately.  Table 1 details the sample of patients and implants.

Clinical Data

A higher mean peri-implant crevicular fluid volume (CFV) was observed in immediate implants (Group A) versus non-immediate implants (Group B) at all study time points, in both maxilla and mandible and overall; while overall mean CFV did not show statistically significant differences between groups, there were significant differences between groups in maxillary CFV at time points 2 and 3 ( Table 2).

**Table 2:**
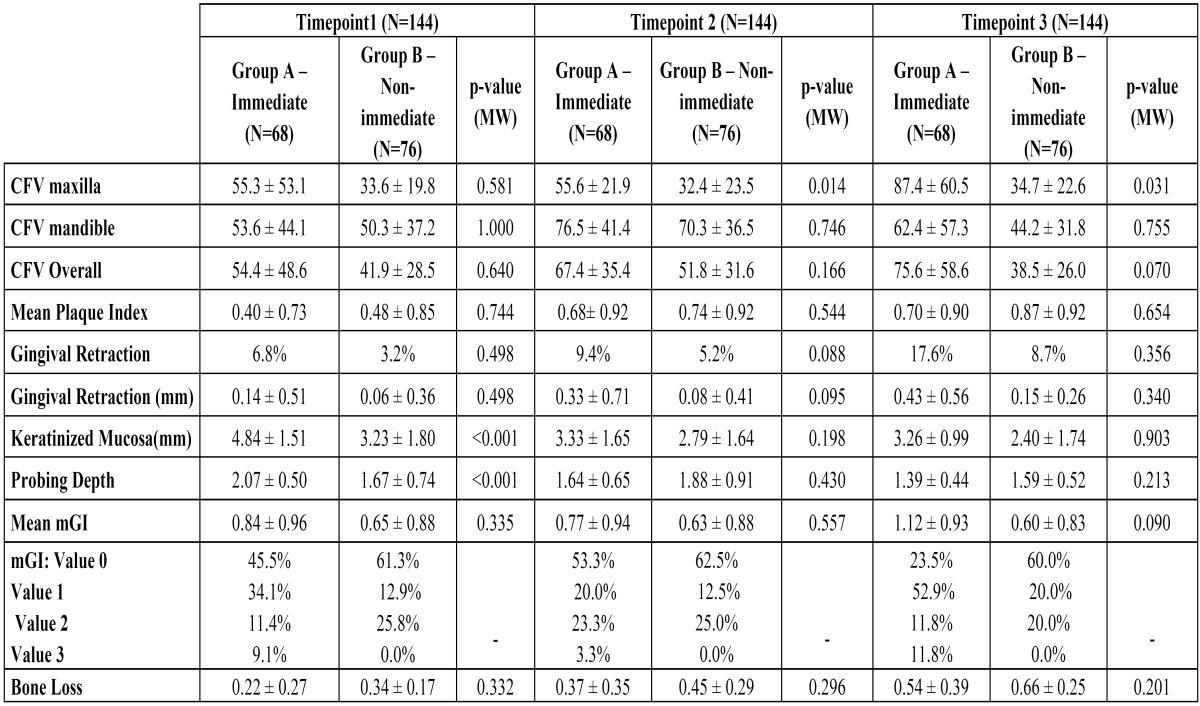
Clinical parameters and radiographic results (marginal bone levels) at each follow-up time point after implant Placement.

Although plaque levels increased along the different time points, no statistically significant differences were identified between groups (*p*≥0.05 - Mann-Whitney U-test) ( Table 2).

Gingival retraction and probing depth increased in both groups over time; no significant differences were observed between the two groups at any of the time points. Keratinized mucosa width was seen to decrease in both groups over time; the group of immediate implants had more keratinized mucosa throughout the study, although differences were only statistically significant at time point 1 ( Table 2).

Modified gingival index was slightly higher among immediate implants (Group A) although no significant differences between groups were identified ( Table 2).

There were no implant failures in either group. The overall implant success rate after the 12-month follow-up was 100% in both groups. All the implants fulfilled the success criteria defined by Buser *et al.* (13) at the 12-month follow-up.

The difference between the average bone loss for immediate implants (Group A: 0.54 ± 0.39 mm) and for those placed in healed sites (Group B: 0.66 ± 0.25 mm) was not statistically significant (*p*=0.201).

## Discussion

The purpose of this study was to evaluate and compare peri-implant health, marginal bone loss and success of immediate and delayed implants rehabilitated with full-arch fixed prostheses. According to a recent literature review (6), to date there have only been a very limited number of randomized clinical trials that compare immediate and delayed implants. No controlled studies have been found evaluating the influence on peri-implant health of placing implants immediately following tooth extraction or after allowing socket healing in patients rehabilitated with fixed full-arch prostheses. Randomized assignment of the selected patients should provide the high-quality evidence for surgical protocols (immediate implant and non-immediate implant placement) that is lacking in the literature. In spite of the reduced sample size - 15 patients and 144 implants (although with a statistical power of 95%) - the present study aimed to add to the available evidence for evaluating and comparing peri-implant health, marginal bone loss and success of immediate and delayed implants rehabilitated with full-arch fixed prostheses; the sample consisted of 15 consecutive patients selected by means of strict, uniform criteria and treated by the same team of professionals using exactly the same procedures.

Investigation of the biochemical parameters in gingival or peri-implant CFV will determine the current activity of the disease, the patient’s susceptibility and possible destruction in the future. So far, numerous studies have focused on CFV analysis in the aim of identifying potential host markers which might provide diagnosis of disease activity and/or prognosis of future disease (14,15). Several studies have shown that CFV volume increases significantly in the presence of inflammatory conditions (16,17) and increased CFV volume is a useful marker of inflammation of periimplant tissue as well as gingival tissue (16). In the present study, higher mean peri-implant CFVs were observed in immediate implants at all study time points, in both maxillae and mandibles and overall. Berglundh *et al.* (18) describe a model for investigation of the different phases of wound healing involved in the processes resulting in osseointegration, concluding that osseointegration represents a dynamic process both during its establishment and its maintenance. In the establishment phase, there is a delicate interplay between bone resorption in contact regions (between the titanium body and mineralized bone) and bone formation in ‘contact-free’ areas. During the maintenance phase, osseointegration is secured through continuous remodeling and adaptation to function. The increase in peri-implant CFV could be due to greater neutrophil and macrophage activity, which participate in the initial phases of osteointegration.

In a recent literature review (19), after reviewing 171 articles, 13 prospective clinical studies on single immediate implant treatment were selected. Midfacial recession was described in 0-64% of the cases. Only one of these studies identified a high risk of advanced midfacial recession (>10%) (20). In the present study, retraction at the 12-month follow-up for Group A immediate implants was 17.6% (0.43±0.56mm) compared to 8.7% (0.15±0.26mm) for Group B implants placed at healed sites. These results coincide with the findings obtained in other studies (21,22), who obtained values ranging between 0.41 and 0.55mm for immediately placed implants.

With respect to plaque index, this increased over the different time points, but without statistically significant difference in either group. These results are slightly higher than those reported by Visser *et al.* (23), who obtained a mean of 0.4 ± 0.8 for non-immediate implants, while in the present study patients with non-immediate implants were rehabilitated with overdentures.

Nishimura *et al.* (4) and Chung *et al.* (24) studied immediate and non-immediate implants, obtaining mean probing depths of 2.3 ± 0.5 mm and 2.86 ± 0.08 mm respectively at the 12-month follow-up; most implants presented peri-implant pockets with depths of less than 3mm. These results are consistent with those obtained in this study: 1.59 ± 0.52 mm in implants at healed sites and 1.39 ± 0.44 mm for immediate implants. Similar results were also reported by Botticelli *et al.* (10), who found a mean probing depth of 2.4 mm for immediate implants and Boynuegri *et al.* (15) who found 1.71 mm for implants at healed sites.

While Wennström *et al.* (25) have reported that keratinized mucosa does not significantly influence oral hygiene status and soft tissue health, Chung *et al.* (24) have found that the absence of adequate keratinized mucosa in dental implants, especially in posterior implants, was associated with higher plaque accumulation and gingival inflammation but not with increased annual bone loss.

In this study, changes in the width of keratinized mucosa were studied in immediate and non-immediate implants; in both groups the width of keratinized mucosa decreased after 12 months but no significant differences were found between groups. According to Boynuegri *et al.* (26) an adequate band of keratinized mucosa was related to less plaque accumulation and mucosal inflammation as well as pro-inflammatory mediators such as IL-1β and TNFα present in CFV. However, the present study findings did not establish this relationship. This could be due to the dynamics of the osseointegration process both during its establishment and its maintenance and to greater neutrophil and macrophage activity in immediately placed implants (18).

 With regard to the bleeding index, Lachmann *et al.* (27), studying non-immediate implants, obtained a mean of 0.4 bleeding on probing with a range from 0 to 2; this value cannot be compared with results obtained in the present study since the present study used a different evaluation system, the Mombelli modified Gingival Index (range 0 to 3). At 12 months after prosthetic loading, the average mGI was 1.12 ± 0.93 for immediate and 0.60 ± 0.83 for non-immediate implants.

Several authors (2) have studied the survival rates of immediate and non-immediate implants, finding no statistically significant differences between the survival rates of immediate and non-immediate implants. These results are consistent with the present study’s findings.

The difference between the average bone loss for immediate implants (Group B: 0.54 ± 0.39 mm) and for those placed in healed sites (Group A: 0.66 ± 0.25 mm) was not statistically significant. Crespi *et al.* (28) evaluated changes in the bone around 40 immediate implants, 20 immediately restored and 20 loaded after three months. The authors reported a success rate of 100% in both groups, and radiographic results were similar. A review by den Hartog *et al.* (29) of single implant restorations in the esthetic zone, found no statistically significant differences in clinical trials comparing immediate, early and delayed implant placement. In contrast, for Atieh *et al.* (30) there is an added risk in the immediate restoration of immediate implants placed in the esthetic region compared to those placed in healed sites. However, according to these authors this approach may offer advantages with respect to favorable changes in marginal bone levels, the maintenance of soft tissues around the implant, and better esthetic results. Lindeboom *et al.* (7) compared peri-implant bone loss around immediate and non-immediate implants and found lower losses around immediate implants although differences were non-significant. Botticelli *et al.* (10) treated 18 patients with 21 immediate implants and after a follow-up of five years found stable bone levels and even gains around some implants.

The present study with a short follow-up and a small sample yielded no statistically significant differences in implant success and peri-implant marginal bone loss between immediate and non-immediate implants supporting fixed full-arch prostheses. No statistically significant differences were found in peri-implant health for any of the parameters studied (crevicular fluid volume, plaque index, gingival retraction, keratinized mucosa, probing depth, modified gingival index and presence of mucositis) to the twelve months follow-up. Further studies with longer follow-up times and larger samples are required to better evaluate the influence of immediate and non-immediate implants on peri-implant health.
